# PM_2.5_ exposure and anxiety in China: evidence from the prefectures

**DOI:** 10.1186/s12889-021-10471-y

**Published:** 2021-03-02

**Authors:** Buwei Chen, Wen Ma, Yu Pan, Wei Guo, Yunsong Chen

**Affiliations:** 1 Department of Sociology, School of Social and Behavioral Sciences, Nanjing University, Nanjing, 210023 Jiangsu Province China; 2 JD.com Retail, Technology and Data Center, Transaction Product Department, Core Transaction Product Group, Beijing, China; 3 Center on Population, Environment, Technology, and Society (C-PETS), School of Social and Behavioral Sciences, Nanjing University, Nanjing, 210023 Jiangsu Province China

**Keywords:** Anxiety, PM_2.5_, Baidu index, Two-way FE model, China

## Abstract

**Background:**

Anxiety disorders are among the most common mental health concerns today. While numerous factors are known to affect anxiety disorders, the ways in which environmental factors aggravate or mitigate anxiety are not fully understood.

**Methods:**

Baidu is the most widely used search engine in China, and a large amount of data on internet behavior indicates that anxiety is a growing concern. We reviewed the annual Baidu Indices of anxiety-related keywords for cities in China from 2013 to 2018 and constructed anxiety indices. We then employed a two-way fixed effect (FE) model to analyze the relationship between PM_2.5_ exposure and anxiety at the prefectural level.

**Results:**

The results indicated that there was a significant positive association between PM_2.5_ and anxiety index. The anxiety index increased by 0.1565258 for every unit increase in the PM_2.5_ level (*P* < 0.05), which suggested that current PM_2.5_ levels in China pose a considerable risk to mental health.

**Conclusion:**

The enormous impact of PM_2.5_ exposure indicates that the macroscopic environment can shape individual mentality and social behavior, and that it can be extremely destructive in terms of societal mindset.

## Background

Anxiety is characterized by inner discomfort, and it is usually accompanied by agitated behavior due to disproportionate concerns over safety, the future, personal fate, and the fates of others [1]. Anxiety affects all populations, and it has long been among the most prevalent and debilitating psychiatric conditions worldwide [2]. Stein et al. (2020) found that anxiety disorders were the sixth leading cause of long-term disability in high-income, low-income, and middle-income countries [3]. Its impacts on health, physical function, and economic output are highly detrimental [4]. Many previous studies on anxiety have focused on the influence of factors at the individual level [5, 6]. However, in his discussion on anxiety research methodology, Hunt (1999) described anxiety as a reaction to dangerous situations and a product of social change [7]. In cultural terms, May (2010) described anxiety as a spreading uneasiness [8].

In times of great social change, individuals who anticipate negative events feel insecure [9]. This type of insecurity is a form of anxiety. Since significant social change affects everyone within a society, anxiety builds at the individual level and eventually affects society at the macroscopic level. Unlike individual anxiety, macroscopic or societal anxiety is a property of societal structure that is broadly distributed among various groups. The influences of social and economic factors on anxiety at the macroscopic level are the most well understood (e.g., Gough, 2009; Viseu et al., 2018) [10, 11], however, environmental factors that aggravate or mitigate anxiety have received less attention. Environmental pollution is among the most serious problems facing populations worldwide. Particulate matter in the atmosphere with diameters of 2.5 μm or less are referred to as PM_2.5_, which is an important measure of air pollution and haze [12]. PM_2.5_ is a health hazard because it can readily enter the lungs [13–15]. PM_2.5_ exposure has been linked to changes in the central nervous system associated with mental disorders [16–18]. In support of the Air Pollution Prevention and Control Action Plan promulgated by the State Council of China in 2013, a series of stringent clean air actions was implemented from 2013 to 2017. With the implementation of stringent clean air actions, PM_2.5_ concentration across the country decreased rapidly [19]. Given the issues mentioned above, we investigated whether PM_2.5_ levels had a direct impact on societal anxiety in the contexts of gross domestic product (GDP), housing prices, the rate of urbanization, internet development, medical resource, and health service level.

China has experienced rapid economic development and monumental social change. However, the health and longevity of Chinese citizens have not increased significantly [20]. The incidence of mental illness has increased markedly along with rapid economic development [21]. Anxiety in particular has become a common social phenomenon. Huang et al. (2019) confirmed that the prevalence of anxiety disorders in China was 4.98% in 2017, which was significantly higher than it was in surveys conducted in the 1980s and 1990s [22]. Anxiety is often stigmatized in China, which makes affected individuals unwilling to reveal their psychological status. Thus, using traditional survey methods and clinical protocols to assess the extent of anxiety in China would likely yield inaccurate results. Analyzing internet search behavior to detect anxiety has unique advantages over traditional survey methods and medical tests. A research subject can privately search for relevant information in the absence of a third party, which dramatically reduces underreporting relative to traditional survey methods. Similar methods are often used to reveal attitudes or behaviors that are difficult to capture in an academic setting [17, 18, 23]. Baidu is the most prevailing Web search engine in China with over 80% of market share [24]. We compiled a list of anxiety-related terms used for internet searches on Baidu. The wide use of Baidu in China therefore makes it a more representative search query for using the terms to construct anxiety indicators. We then examined whether and how local PM_2.5_ levels were associated with anxiety levels using a nationally representative panel of data collected in 297 Chinese cities in the years from 2013 to 2018.

## Methods

### Data

The data used for this analysis was obtained from multiple sources. Baidu search terms submitted in the years from 2013 to 2018 were used to construct anxiety indices for cities at the prefectural level. We collected average monthly PM_2.5_ data from China’s Air Quality Online Monitoring and Analysis Platform and used them to calculate the annual PM_2.5_ levels.^1^ Data about economic and urban development in the years from 2013 to 2017 were obtained from the statistical yearbooks of Chinese cities, while the 2018 data were obtained from provincial statistical yearbooks. Data about medical resource and health service level in the years from 2013 to 2018 in this research were obtained from the China health statistics yearbook. We then constructed a representative panel of data for 293 prefecture-level cities and four municipalities for the years from 2013 to 2018. The total sample size was 1782.

### Measures

#### Anxiety index

We first constructed a list of words related to anxiety (Appendix, Table 3). The selection of words was based on life experience and the Baidu demand map,^2^ which showed terms that frequently appeared together with “anxiety” in online searches. These terms were related to specific psychological and social pressures that could cause anxiety. For example, individuals usually associate hair loss (tuo fa) with anxiety due to the stigmatization of baldness. Internet search behavior thus reflected the psychological state of the individual and the influence of negative social attitudes.

The Baidu Index is the official database and data-sharing platform of the Baidu search engine, and it reflects the behavior of Baidu users. The Baidu Index provides detailed information about keywords that appear in internet searches. We collected the annual Baidu Indices for all keywords entered by internet searchers in each city in the years from 2013 to 2018 and standardized the indices by including each anxiety-related keyword. The anxiety index for each city was the ratio of the Baidu Index summation to the city’s population. We could review data for a given day, week, month, or year at both the provincial and national levels. Selecting keywords for online searches is an active and problem-driven process. Although we could not measure anxiety directly, the keywords were subjective choices based on concerns about specific problems. Users may have been experiencing such problems themselves, or they may have had friends or relatives with psychological issues. This motivated users to “Baidu it” to obtain explanation or relief.

The internet users comprised only a portion of the Chinese population, therefore sampling bias was inevitable. However, Baidu has a wide range of users, and the data reflects real online search behavior. We thus concluded that the data was sufficiently representative for our research.

#### Explanatory variables

PM_2.5_ was the key explanatory variable in this study. Others have investigated the relationship between pollution and health, but the relationship between PM_2.5_ and anxiety is an important issue. We controlled for related variables, particularly variables associated with the economy and urban development. The GDP is the sum of all productive activity in a region over a given period of time based on market prices. We used the annual GDPs of prefecture-level cities as indicators of economic development. The GDP of each city encompassed output in administrative regions, urban areas, municipal districts, and the county. We evaluated the per-capita GDPs and obtained similar results.

Soaring housing costs in first- and second-tier cities are a source of anxiety for many residents [25–27], therefore we also used the average annual housing price in each prefecture-level city as an indicator. The housing data can be found at gotohui.com,^3^ which is an instrumental platform for housing price queries.

The ratio of the urban population to the total population in a region reflects its degree of urbanization. The urbanization ratio of each prefecture-level city was thus used as an index to quantify urbanization.

Broadband internet access was based on the number of users who subscribed to a telecommunications enterprise at the end of the reporting period. Residents in China can access the internet wirelessly or through XDSL, FTTX+LAN, and WLAN connections. Other ways to access the internet include XDSL, dedicated LAN lines, and LAN end use. The proportion of internet broadband users in the population of each prefecture-level city was used as a measure of internet development. Information about broadband internet access and the average yearly population of each prefecture-level city was obtained from the China City Statistical Yearbook. In addition to these variables of major interest, we also measured medical resource and health service level in local areas using the variables of number of medical institutions of each prefecture-level city, total hospital beds of each prefecture-level city, and number of physicians of each prefecture-level city. The descriptive statistics for the variables are shown in Table 1.

**Table 1:** Descriptive Statistics for Variables

Variable		Mean	S. D.	Minimum	Maximum
Anxiety	overall	−14.36	61.42	− 1070.88	555.21
	between		63.28	− 527.52	170.85
	within		28.5	− 611.8	369.99
GDP (10 million YUAN)	overall	25,260.21	34,796.64	1534.09	326,799
	between		33,197.75	1877.55	252,222.6
	within		9278.67	−38,063.65	102,245
Housing price (YUAN)	overall	7786.84	6278.27	662	58,654
	between		5068.98	3069	46,223.83
	within		2151.64	− 8736.33	22,153.67
Urbanization rate (%)	overall	55.82	14.33	24.69	100
	between		14.1	26.5	99.92
	within		2.38	46.06	72.01
Internet development level	overall	0.22	0.41	0.01	11.21
	between		0.30	0.04	4.41
	within		0.28	−1.66	9.50
PM_2.5_	overall	53.8	28.92	8.58	210
(μg·m^−3^)	between		17.65	9.71	109.61
	within		22.99	6.24	172.05
Number of medical institutions	overall	532.79	1422.40	16.67	50,800
	between		716.09	33.33	10,403.64
	within		1224.46	− 9543.58	40,929.15
Total hospital beds	overall	49,875.04	62,720.37	500	2,411,300
	between		31,345.24	1000	435,039.4
	within		54,456.75	− 353,626.3	2,026,136
Number of physicians	overall	24,855.84	27,461.73	50	934,500
	between		16,064.50	91.67	165,586.6
	within		22,415.29	− 132,360.9	793,769.2

#### Modeling strategy

We employed a two-way fixed effect (FE) regression model to examine how PM_2.5_ and related macroscopic factors might affect anxiety in China. The two-way fixed effects regression model with unit and time fixed effects is a default methodology for estimating causal effects from panel data and adjusting for unobserved unit-specific and time-specific confounders at the same time [28]. Therefore, this made it easy to rule out any confounding effects from time-invariant factors at the city level in our study. The model is represented by Eq. 1.

$$ {\displaystyle \begin{array}{l}{\mathrm{Anxiety}}_{it}={\beta}_0+{\beta}_1{\mathrm{GDP}}_{it}+{\beta}_2{\mathrm{price}}_{it}+{\beta}_3{\mathrm{urban}}_{it}+{\beta}_4{\mathrm{internet}}_{it}+\\ {}{\beta}_5\mathrm{PM}2{.5}_{it}+{\beta}_6{\mathrm{institution}}_{it}+{\beta}_7\kern0.5em {\mathrm{bed}}_{it}+{\beta}_8{\mathrm{physician}}_{it}+{\upsilon}_i+{\delta}_{it}\\ {}i=1,2,\dots \dots, \mathrm{n};t=1,2,\dots \dots, \mathrm{T}\end{array}} $$

where Anxiety_*it*_ is a dependent variable that represents the level of anxiety in city *i* at time *t*. The larger the anxiety index, the higher the level of anxiety. GDP is the regional gross domestic product, which reflects the level of urban economic development. *Price* represents the average annual cost of housing in the city; *Urban* is its urbanization level; and *Internet* represents the level of internet development in the city. PM_2.5_ is the key independent variable, which represents the annual PM_2.5_ level in city *i* in year *t*. *Institution, bed, and physician* represent the number of medical institutions, total hospital beds, the number of physicians in the city, respectively. The random variable *υ*_i_ is an unobservable effect term that reflects individual heterogeneity, and *δ*_it_ is a disturbance term that varies over time.

## Results

### Spatiotemporal trends in anxiety

**Fig. 1:**
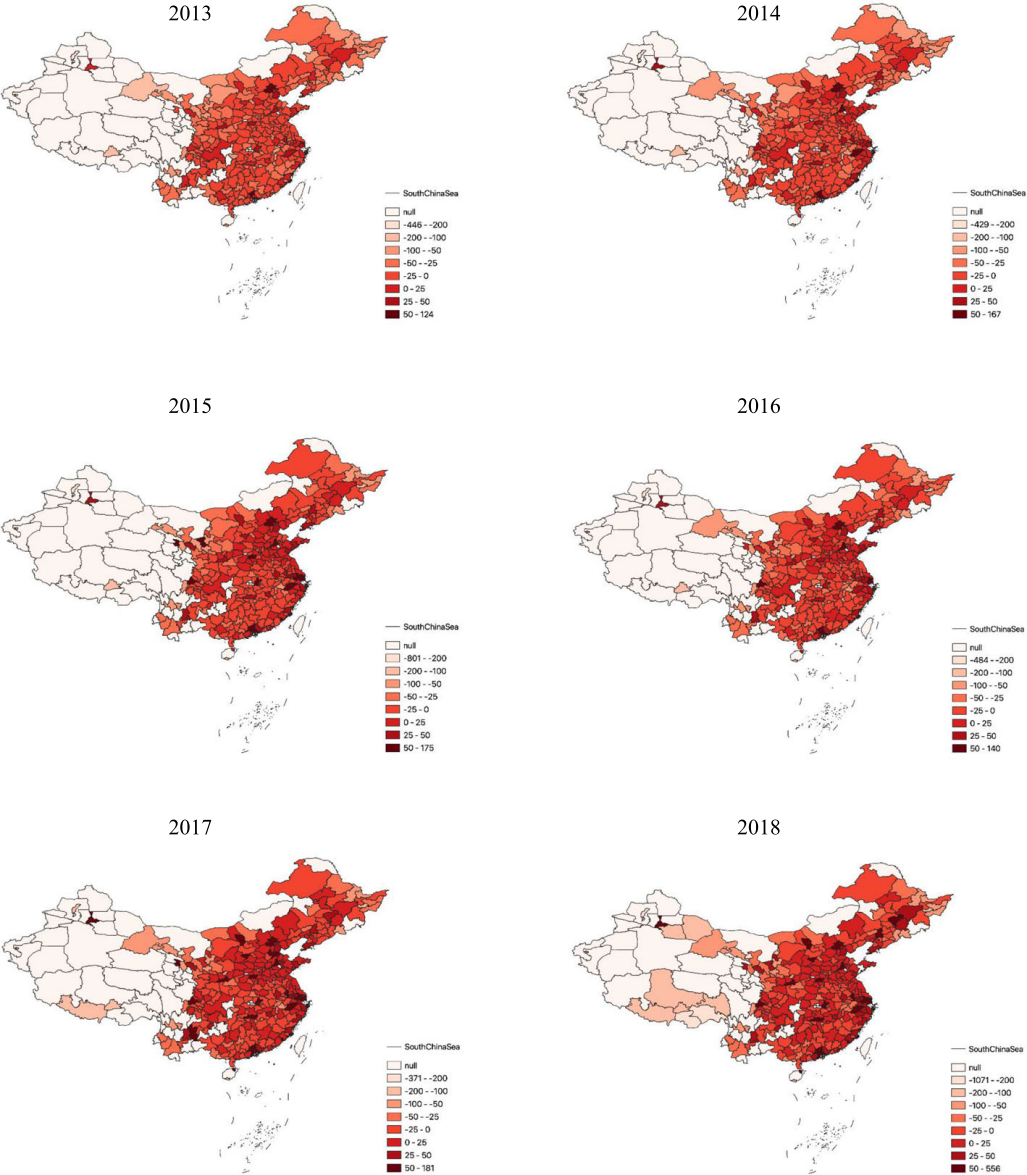
Spatial Distributions of Anxiety Indices in China. *Note.* The maps are generated using GIS 10.5

Anxiety in China fluctuated, but it followed an increasing trend overall. The anxiety index was approximately 2.7-fold higher in 2018 than it was in 2013. The spatial distributions of anxiety in the years from 2013 to 2018 are shown in Fig. 1. The anxiety indices of Guangdong Province in southern China, Shanghai, Jiangsu, and Zhejiang in eastern China were the highest in the country. The anxiety indices of Beijing and Tianjin were also relatively high.

In 2013, anxiety was either severe or moderate in 40 Chinese cities (Fig. 1). The number of coastal cities with moderate anxiety continued to increase in 2014, and moderate anxiety was detected in a small number of inland cities. By the end of 2014, a total of 65 cities were severely or moderately anxious. Anxiety was moderate or severe in 98 cities in 2015. Moderate anxiety continued to increase in coastal areas, and more inland cities north of the Yangtze River appeared to be moderately anxious. In 2016, severe or moderate anxiety was detected in 88 coastal cities. While the number of cities with moderate anxiety decreased overall, the number of inland cities with moderate anxiety rose slightly. A total of 137 cities experienced severe or moderate anxiety in 2017, and the number of moderately anxious cities in coastal areas increased. Moderate anxiety was also detected in cities in central and southwest China. A total of 139 cities experienced severe or moderate anxiety in 2018. The number of coastal cities with moderate anxiety fell slightly, but anxiety continued to increase in the southwestern and southern regions.

### Results of two-way FE modeling

The two-way FE modeling results are shown in Table 2. Our results were not consistent with those of a previous study [25], and there was no significant association between housing cost and the anxiety index. There was significant correlation between the anxiety index and the urbanization rate (*P* < 0.1). One-unit increases in the urbanization rate was associated with a − 1.97668 decrease in the anxiety index. After controlling for other factors, we found that the anxiety index decreased by 0.0009126 for every unit increase in hospital beds.

**Table 2:** Results of Two-way FE Modeling

Variable	Coeff.	S. E.	T
GDP	−0.0000995	0.0001003	−0.99
Housing price	0.0005113	0.0003328	1.54
Urbanization rate	−1.97668^*^	1.002774	−1.97
Internet development level	19.10118	15.85514	1.2
Number of medical institutions	−0.0007544	0.0029432	−0.26
Total hospital beds	−0.0009126^*^	0.0003858	−2.37
Number of physicians	0.0022751^*^	0.0009954	2.29
PM_2.5_	0.1565258^**^	0.0576524	2.71
year2	22.53083^***^	4.218837	5.34
year3	35.07602^***^	5.890775	5.95
year4	34.12029^***^	6.943305	4.91
year5	37.6161^***^	7.326584	5.13
year6	43.60162^***^	9.2261	4.73
R^2^ (Between Groups)	0.0155		
R^2^ (Within Groups)	0.6255		
R^2^ (Overall)	0.0008		
F	27.14 (Prob > F = 0.0000)		
N	928		

Notes: *P < .05; **P < .01; ***P < .001

The model also demonstrated that PM_2.5_ exposure was positively and significantly associated with the anxiety index. The anxiety index increased by 0.1565258 for every unit increase in the PM_2.5_ level (*P* < 0.05). According to China’s Air Quality Online Monitoring and Analysis Platform, it is quite normal for the PM_2.5_ level to increase from 50 μg·m^− 3^ to 100 μg·m^− 3^ within one week. Our results indicated that an increase of this magnitude would raise the anxiety index by approximately eight units. The effect of PM_2.5_ exposure on anxiety levels was thus considerable, and it impacted the anxiety index to a much greater extent than other related factors.

## Discussion

Anxiety is becoming increasingly common in China as social transition progresses. We examined changes in urban anxiety and the social and environmental factors that influenced it during social transition in China. We used our Baidu Index of anxiety-related words to construct the anxiety indices and included information about the GDPs, housing costs, urbanization rates, internet development levels, medical institutions, total hospital beds, the number of physicians, and PM_2.5_ levels in 297 cities in the years from 2013 to 2018 to develop a representative model.

Urban anxiety fluctuated, but the observed trend was an overall increase. The number of cities with moderate anxiety continued to increase over time. Cities with anxiety indices greater than zero experienced either moderate or severe anxiety, and the number of inland cities in this category gradually increased. Two-way FE modeling revealed that PM_2.5_ levels were significantly and positively correlated with anxiety. After controlling for related factors, we found that the anxiety indices rose with increases in PM_2.5_ index. PM_2.5_ levels had a much stronger influence on the anxiety indices, which indicated that PM_2.5_ was the most important contributor to anxiety.

Studying anxiety at the macroscopic level has great practical value. The social structure of China is undergoing significant changes, and social priorities are shifting. Access to education, medical care, pensions, and social security have become sources of anxiety.^4^ Understanding anxiety in this context will facilitate smooth social transition in China. Social transition involves transformations at both the material level and in social mentality. A positive social mentality will be conducive to positive social change, while a negative social mentality will have adverse effects. Crime and violence are more likely when anxiety is a common societal affliction [29]. Thus, relieving anxiety serves a vital purpose in promoting positive and healthy social development.

Using big data to develop indices for anxiety measurement was an innovative way to compensate for the deficiencies of existing methods used to measure anxiety. Zhou (2014) has shown that anxiety is a type of social mentality with emerging properties [30]. In other words, anxiety at the macroscopic level arises from anxiety at the individual level. However, societal anxiety cannot be reduced to the individual level, because it has unique characteristics and functions. Wilkinson (2001) states that when individual anxiety becomes universal, it will cause social tension and negatively impact smooth societal operation [31]. A problem with current anxiety research is that theoretical analyses are much more common than empirical studies. Empirical studies often assume that a coalescence of personal anxiety represents societal anxiety. However, the explanatory power of macroscopic anxiety is reduced when it is defined this way. To address this problem, we compiled a Baidu Index of anxiety-related words to represent anxiety. The index reflected both individual anxiety and the impact of individual stress as a social phenomenon. Constructing an anxiety index this way provided a better representation of anxiety at the macroscopic level. Current empirical research primarily addresses stress in specific groups based on the answers to questionnaires. Only a few studies have been conducted from a macro-sociological perspective, and most of them have been theoretical in nature. To perform an empirical analysis with a macroscopic focus, we analyzed macro-socioeconomic factors that affected anxiety and examined them by constructing a panel model.

### Limitations

The limitations of this study warrant discussion, and they provide direction for future research in this area. Although Baidu is the most popular search engine and accounts for more than 80% search market share in China, we acknowledge that the proportion of Baidu coverage across different areas in China might affect the results of our study. The factors that influence societal anxiety include transitions in social structure, social risk, social security, political reform, modern technology, and cultural values. We were limited by the amount of available data and the difficulty of identifying influencing factors. Thus, we only considered PM_2.5_, GDPs, housing costs, urbanization rates, levels of internet development, medical resource, and health service level. Since we studied short-term anxiety over five years, some of the macroscopic factors related to crisis and reform may not have had much impact on anxiety. This limitation influenced our selection of measures in this study. Although anxiety at the macroscopic level can be assumed to have characteristics that differ from individual anxiety, internet users do not represent all members of society.

## Conclusion

Despite its limitations, our analysis has generated some intriguing questions about quantifying anxiety and using large datasets to study macroscopic influences. Among the macroscopic factors we examined, PM_2.5_ was the most important atmospheric pollutant associated with anxiety. We used PM_2.5_ levels as air pollution indicators and analyzed the relationship between PM_2.5_ exposure and anxiety. We found that PM_2.5_ exposure and anxiety were significantly correlated. Anxiety is a condition that affects society as a whole. The enormous impact of PM_2.5_ exposure indicates that the macroscopic environment can shape individual mentality and social behavior, and that it can be extremely destructive in terms of societal mindset. Pollution is a great societal hazard in both spiritual and material terms. Analyzing the associations between air pollution and anxiety in more detail could provide solutions to alleviate anxiety in China.

## Data Availability

All data are available from the corresponding author on reasonable request.

## Appendix

**Table 3:** List of Anxiety-related Words

Number	Word
1	Anxiety
2	Anxiety disorder
3	Anxious neurosis
4	Depression
5	Melancholia
6	Depressive neurosis
7	High pressure
8	High pressure (There is another expression in Chinese)
9	Hair loss
10	Nervous
11	Insomnia
12	Feeling of anxiety
13	Depression test
14	Depression test (There is another expression in Chinese)
15	Pressure test
16	Hairline moving back
17	Worry
18	Mental stress
19	Occupational stress
20	Psychological pressure
21	Stress management

Notes: Internet user search messages in Baidu using Chinese and the translation of each Chinese keywords is listed in English

## Abbreviations

FE: Fixed effect
GDP: Gross domestic product
DSL: Digital subscriber line
WLAN: Wireless local area networks

## Footnotes

1 See https://www.aqistudy.cn

2 See https://index.baidu.com/v2/main/index.html#/demand/焦虑?words=焦虑

3 See https://www.gotohui.com

4 https://news.gmw.cn/2019-08/22/content_33097077.htm
